# Spatial distribution of hyperpolarized [1-^13^C]pyruvate MRI and metabolic PET in the human brain

**DOI:** 10.1162/IMAG.a.903

**Published:** 2025-09-26

**Authors:** Tyler Blazey, Andrei G. Vlassenko, Manu S. Goyal, Hany Soliman, Charles H. Cunningham, Cornelius von Morze

**Affiliations:** Mallinckrodt Institute of Radiology, Washington University School of Medicine, St. Louis, MO, United States; Department of Neurology, Washington University School of Medicine, St. Louis, MO, United States; Department of Neuroscience, Washington University School of Medicine, St. Louis, MO, United States; Radiation Oncology, Sunnybrook Health Science Center, Toronto, ON, Canada; Physical Sciences, Sunnybrook Research Institute, Toronto, ON, Canada; Department of Medical Biophysics, University of Toronto, Toronto, ON, Canada

**Keywords:** brain imaging, energy metabolism, MRI, Lactate, positron emission tomography

## Abstract

Magnetic resonance imaging (MRI) of hyperpolarized (HP) [1-^13^C]pyruvate is a promising method for measuring cerebral energy metabolism *in vivo*. The substantial increase in signal provided by HP makes it possible to dynamically monitor the conversion of [1-^13^C]pyruvate to [1-^13^C]lactate and [^13^C]bicarbonate. The HP [1-^13^C]lactate signal is commonly associated with glycolic activity, whereas [^13^C]bicarbonate, a by-product of the reaction that forms acetyl-CoA, is linked to oxidative metabolism. However, there is compelling evidence that other factors, such as the concentration of monocarboxylate transporters, influence the production of HP [1-^13^C]lactate. To clarify the processes responsible for producing the topography of HP [1-^13^C]pyruvate and its metabolites, we spatially correlated group-average HP ^13^C MRI images with [^18^F]FDG, [^15^O]H_2_O, [^15^O]O_2_, and [^15^O]CO positron emission topography (PET) images from a separate group of 35 age- and sex-matched adults. We found that [1-^13^C]pyruvate correlated best with cerebral blood volume (CBV), whereas [1-^13^C]lactate and [^13^C]bicarbonate were most strongly associated with cerebral blood flow (CBF), glucose consumption (CMRglc), and oxygen metabolism (CMRO_2_). Neither [1-^13^C]lactate nor [^13^C]bicarbonate was correlated with non-oxidative glucose consumption, also known as aerobic glycolysis. These results are consistent with the view that in the healthy brain, the production of [1-^13^C]lactate reflects overall energy metabolism rather than being specific to glycolysis.

## 1 Introduction

Positron emission tomography (PET) has long been the reference-standard for imaging energy metabolism in the human brain. Its ability to trace metabolic pathways through radiolabeled compounds makes it a highly specific and flexible technique. Also due to its high sensitivity, PET requires only minimal doses, ensuring that basal metabolic processes are not disrupted by high concentrations of external substances. Finally, PET can yield absolute measurements of metabolic activity when used with an appropriate kinetic model.

Despite these advantages, the radiation exposure associated with PET restricts its use in certain populations (e.g., children), and limits the number of measurements that can be performed in a single individual. It is also not possible with PET to discriminate downstream metabolites of the original tracer, as only the spatial distribution of the radiolabel can be tracked. However, several magnetic resonance imaging (MRI) techniques have been developed to overcome these limitations. Among the most promising of these techniques are magnetic resonance spectroscopic imaging (MRSI) (Bogner et al., 2021), deuterium metabolic imaging (DMI) (De Feyter et al., 2018), and hyperpolarized (HP) ^13^C MRI (Wang et al., 2019). The primary advantages of MRSI are that it does not require a contrast agent and can be performed using standard MRI equipment. However, detecting many important metabolites, such as lactate, is challenging because they are present in small amounts and are obscured by multiple factors, including other closely resonating signals. DMI overcomes this limitation by administering a substantial quantity of a deuterated substance, usually glucose, whose metabolic products can be detected directly using ^2^H imaging (De Feyter et al., 2018) or indirectly by ^1^H MRI (Niess et al., 2023). Although DMI is a promising technique, it typically requires long scan times, making it less well-suited for monitoring temporal dynamics or performing multiple measurements in the same individual during the same scanning session.

HP ^13^C MRI is an emerging medical imaging modality that takes advantage of hyperpolarization techniques, such as dynamic nuclear polarization (DNP), to increase the nuclear polarization of ^13^C-enriched compounds by up to five orders of magnitude (Ardenkjaer-Larsen et al., 2003). As the signal detectable by MRI is proportional to nuclear polarization, hyperpolarization dramatically increases the available MRI signal. This enables *in vivo* metabolic imaging at a temporal and spatial resolution that would not be possible using conventional, thermally polarized, MRI (Golman et al., 2006).

Currently, the most widely used HP ^13^C MRI agent is [1-^13^C]pyruvate. The success of HP [1-^13^C]pyruvate is largely due to its long T_1_ relaxation time (~70 s at 3T), and its rapid conversion to metabolites with distinct chemical shifts. The primary downstream products of [1-^13^C]pyruvate in the human brain are [1-^13^C]lactate and [^13^C]bicarbonate (Grist et al., 2019; Lee et al., 2020). Generally, the [1-^13^C]lactate signal is thought to reflect endogenous lactate production (i.e., glycolysis), while [^13^C]bicarbonate, a byproduct of the reaction that produces acetyl-CoA, is considered an index of oxidative metabolism (Wang et al., 2019).

However, because HP MRI requires a relatively large dose of [1-^13^C]pyruvate (0.1 mmol/kg) and measurements are made over a short time window (~1 min), there is good reason to believe that the HP [1-^13^C]lactate signal reflects metabolic processes in addition to lactate production. Evidence from cell lines, animal models, and human studies suggests that the pre-existing lactate concentration (Day et al., 2007; Hurd et al., 2013) and the expression level of monocarboxylate transporters (Granlund et al., 2020; Rao et al., 2020) influence HP [1-^13^C]lactate production. Interestingly, multiple groups have also provided evidence that [1-^13^C]lactate is shuttled to oxidative pathways in both rodent (Bøgh et al., 2022) and human (Uthayakumar et al., 2024; Zaidi et al., 2024) brain.

The aim of this study is to investigate the metabolism of HP [1-^13^C]pyruvate in the human brain by comparing its regional distribution with four different metabolic PET tracers from a separate age- and sex-matched group of adults (Goyal et al., 2023). Specifically, we used [^15^O]H_2_O for cerebral blood flow (CBF), [^18^F]FDG for cerebral glucose consumption (CMRglc), [^15^O]O_2_ for cerebral oxygen consumption (CMRO_2_), and [^15^O]CO for cerebral blood volume (CBV). From these tracers we also derived the oxygen extraction fraction (OEF) and the glycolytic index (GI). OEF indicates the fraction oxygen of extracted from the arterial supply in a single circulatory pass, while GI quantifies aerobic glycolysis, or the consumption of glucose in excess of oxygen consumption.

## 2 Methods

### 2.1 Datasets

The previously published HP ^13^C MRI dataset consisted of 35 cognitively normal participants (14 Male, 21 Female) with ages ranging from 21 to 77 years (Mean = 38, SD = 18). Following the injection of 0.43 mL/kg of 250 mM HP [1-^13^C]pyruvate, ^13^C imaging was performed using a 3D dual-echo echo-planar imaging (EPI) sequence (Geraghty et al., 2018) on a GE MR750 3.0 T scanner. A dual-echo readout (ΔTE = 764 ms) was chosen for its greater signal-to-noise ratio (SNR) compared to a single-echo readout, and because phase differences between the two echoes can be used for off-resonance correction (Geraghty et al., 2018). The EPI sequence acquires whole-brain (FOV 240 x 240 x 360 mm^3^) images of [1-^13^C]pyruvate, [1-^13^C]lactate, and [^13^C]bicarbonate every 5 s with 15 mm isotropic voxels. Each 3D-volume was acquired using 24 excitations, with a net flip angle of 11° for [1-^13^C]pyruvate and 80° for [1-^13^C]lactate and [^13^C]bicarbonate. A total of 12 images were acquired for each metabolite over the 60s acquisition window. Participants were not instructed to keep their eyes open or closed. All ^13^C images were acquired using a custom 8-rung birdcage coil (26 cm diameter, 25 cm long). This coil was removed after ^13^C imaging and replaced with an 8-channel ^1^H coil (Invivo Inc. Pewaukee, WI) to acquire a high-resolution T1-weighted (T1w) image (1 mm isotropic voxels, 256 mm^2^ FOV, TR 7.6 ms, TE 2.9 ms, flip angle 11°) for anatomical reference. Full details of the image acquisition procedures can be found in the original report (Uthayakumar et al., 2023).

The PET dataset comprised 35 age (Mean = 38 years, SD = 18 years) and sex (14 Male, 21 Female) matched PET participants from the Aging Metabolism & Brain Resilience study (Goyal et al., 2023). Four different radiotracers were administered to each subject during a single visit: 1) 60 mCi of [^15^O]O_2_ for CMRO_2_, 2) 75 mCi of [^15^O]CO for CBV, 3) 50 mCi of [^15^O]H_2_O for CBF, and 4) 5 mCi of [^18^F]FDG for CMRglc. This allowed for a more comprehensive characterization of cerebral metabolism than using a single tracer and allowed us to estimate cerebral oxygen extraction and non-oxidative glucose use (Vaishnavi et al., 2010). All images were acquired on a Siemens ECAT EXACT HR 47 PET scanner, which has a transverse resolution of 3.8–5.0 mm full width at half maximum (FWHM) and an axial resolution of 4.7–5.7 FWHM. Excluding the [^15^O]CO scan, which was collected using a 5-min static scan, all PET data were acquired in dynamic mode. The [^15^O]O_2_ and [^15^O]H_2_O scans lasted for 3 min, while the [^18^F]FDG scan was acquired over 1-h. Participants were asked to keep their eyes closed and to remain awake. As with the HP dataset, high-resolution (1 mm isotropic voxels, 256 mm^2^ FOV) T1w images were acquired for anatomical reference. A more detailed explanation of the PET acquisition protocol can be found elsewhere (Goyal et al., 2017, 2023).

Written informed consent was obtained for all participants, and experiments were approved by the appropriate institutional review board (HP dataset: Research Ethics Board of Sunnybrook Health Sciences Centre, PET dataset: Human Research Protection Office at Washington University in St. Louis).

### 2.2 Image processing

To facilitate regional comparisons across modalities, a group median image was computed for each tracer/metabolite in MNI152 space (Evans et al., 2012). First, the dynamic MRI/PET data for each participant were integrated across time to produce a single sum image. Although HP ^13^C MRI is often analyzed using a kinetic model (Zierhut et al., 2010), most models employ considerable simplifications (e.g., no input function (Mammoli et al., 2020)) and give similar results to temporal summations (Hill et al., 2013). All twelve timepoints of the HP data were integrated, while the PET integration window was tracer specific. A 60 s window starting when the whole-brain activity curve first increased was used to compute CMRO_2_ from [^15^O]O_2_ and CBF from [^15^O]H_2_O, while the last 20 min of the scan was used to obtain CMRglc from the [^18^F]FDG scan. As only a single time-point was collected for [^15^O]CO, no summation was necessary to obtain a CBV image. Vascular artifacts were removed from the CMRO_2_ image by spatial regression with the CBV image.

Each sum image was then aligned to the participant’s T1w image using a rigid body transformation. FSL’s FLIRT was used for registering images from the HP dataset (Jenkinson et al., 2012), while the PET dataset was registered using in-house tools (Rowland et al., 2005). Next, a nonlinear transformation was computed between each participant’s T1w image and the MNI152 template using ANTs (Avants et al., 2011). The two transformations were concatenated, and each sum image was resampled into MNI152 space with 3 mm isotropic voxels. The individual sum images were normalized so that the whole brain (defined using the MNI152 brain mask included in FSL) had a mean of 1. Whole-brain normalization reduces between-subject variance, such as differences in polarization or radiation dose, but limits analyses to regional (i.e., local-to-global) effects. Example HP and PET images from a randomly chosen subject can be found in Supplementary Figures S1 and S2. After normalization, a group median was computed for each tracer/metabolite. Finally, regional medians were obtained for each sum image using the Schaeffer 7 network atlas, which parcellates the cerebral cortex into 200 individual parcels (Schaefer et al., 2018).

Two derivative images were computed from each participant’s PET data. First, OEF was computed as ratio of the [^15^O]O_2_ and [^15^O]H_2_O sum images, after first regressing out the [^15^O]CO image from the [^15^O]O_2_ data. As with the other PET images, the OEF data were normalized to a whole-brain mean of 1 prior to computing the group average image. The glycolytic index (GI), a PET-derived index of aerobic glycolysis (AG), was calculated by taking the residuals of a regression between CMRO_2_ (independent variable) and CMRglc (dependent variable) across the whole brain (Vaishnavi et al., 2010). As a result, GI has a whole-brain mean of 0, with positive values indicating regions where glucose consumption exceeds oxygen consumption, relative to the rest of the brain.

Analogous to GI, we also used linear regression to create HP residual images. For example, the lactate-bicarbonate residual image, (LBR), was computed by taking residuals of a regression between each participant’s [1-^13^C]lactate (dependent) and [^13^C]bicarbonate (independent variable) sum image. The resulting image indicates regions where the [1-^13^C]lactate signal is higher/lower than what would be expected given the [^13^C]bicarbonate signal (Supplementary Fig. S3). The same process was used to generate lactate-pyruvate (LPR) and bicarbonate-pyruvate (BPR) residual images. Although it is commonplace to use ratios to normalize HP images by another metabolite (e.g., lactate-to-bicarbonate ratio), we chose residuals because they eliminate the need for noisy voxelwise division.

Due to the coarser spatial resolution of the HP ^13^C images, the group-average PET images were smoothed with a 14 mm FWHM 3D gaussian kernel. The size of the kernel was empirically chosen to maximize the correlation between the two datasets (Supplementary Fig. S4).

### 2.3 Statistics

The Spearman rank order correlation coefficient, ρ, was used to evaluate the similarity between images. Correlations were computed using all voxels within the MNI152 brain mask included in FSL. However, statistical significance was not assessed at the voxel level because the spatial autocorrelation between voxels would substantially inflate *p*-values (Alexander-Bloch et al., 2018). Instead, significance was determined using the 200 parcel averages. A null distribution was created using the spatial permutation method introduced by Alexander-Bloch et al. (2018). Each permutation consisted of randomly rotating the parcel data on a spherical representation of the cortical surface, and then correlating the rotated data with the unrotated data of the target image. A total of 10,000 permutations were performed using the neuromaps library (Markello et al., 2022). Comparisons with a *p*-value less than 0.05 after correction for multiple regional tests using the false discovery rate were considered significant (Benjamini & Yekutieli, 2001).

Partial least squares (PLS) was used to identify the linear combinations of the HP ^13^C and PET images which maximizes the covariance between the two datasets (Wegelin, 2000). Model fitting was done using ‘PLSCanonical’ function in scikit-learn (Pedregosa et al., 2011). Only the first two combinations, or components, were analyzed. Each component consists of a set of weights that transforms the image data to a set of latent variables, or scores. The magnitude of the weights indicates the strength of association between each input image (e.g., [1-^13^C]pyruvate) and the extracted component. Note that terminology for describing PLS varies, and what we refer to as weights is called rotations in scikit-learn. For visualization purposes, the spatial map for each score was normalized to an absolute maximum of 1.

All plots were created using open-source Python tools (Harris et al., 2020; Hunter, 2007; Virtanen et al., 2020). Every voxel in voxelwise scatter plots was colored according to its value in the bivariate probability density function to better visualize the relationships between variables. The fastkde library was used to estimate the 2D probability density functions (O’Brien et al., 2014, 2016).

## 3 Results

**Fig. 1.**
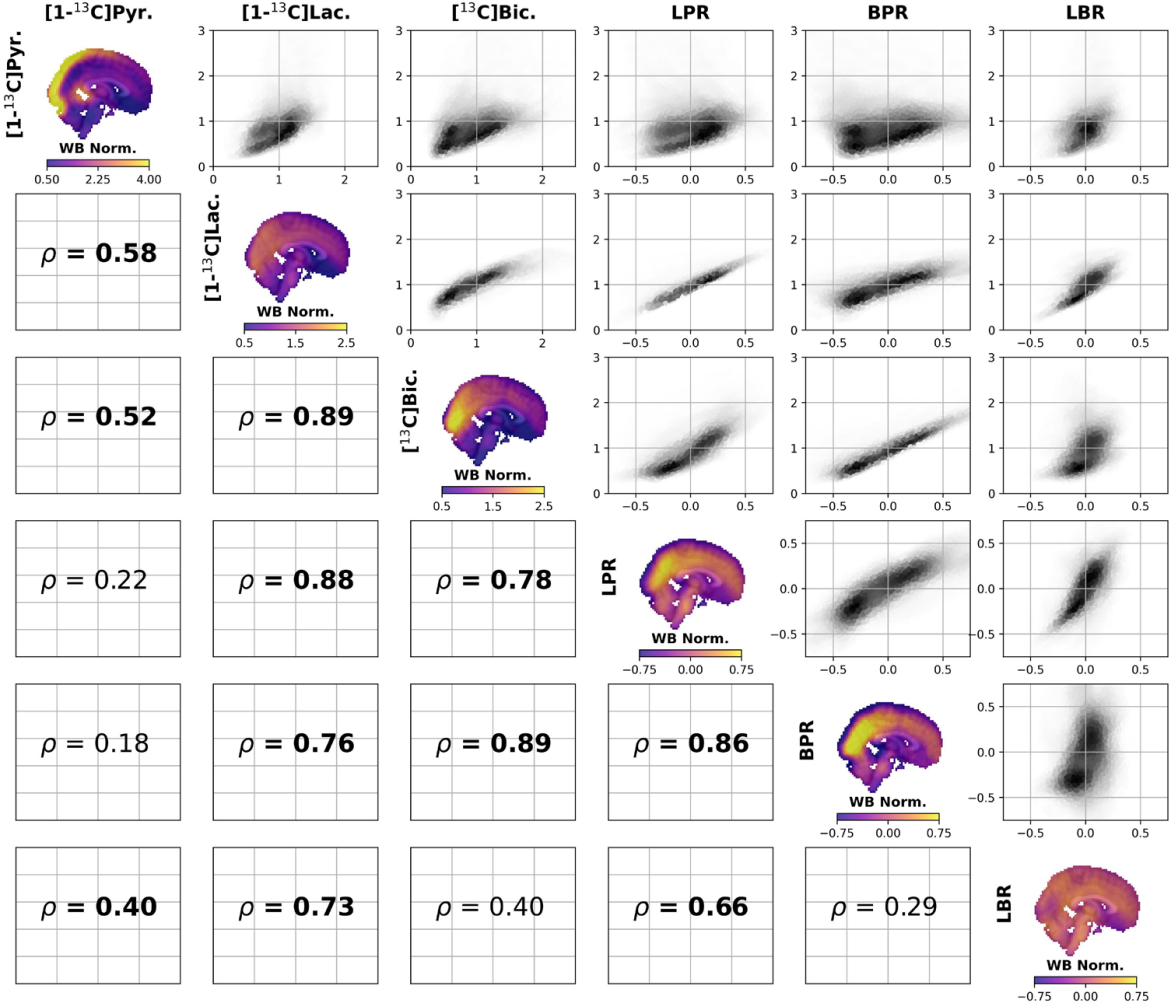
Within-modality comparison of topographies of cerebral metabolism measured with HP ^13^C MRI. On the diagonal is the group-average for each metabolic image. Each cell in the upper triangle is a scatter plot comparing the images in the row/column. The Spearman rank order correlation coefficient for each comparison is shown in the lower triangle, with significant comparisons (*p* < 0.05, FDR corrected) shown in bold (see section 2). A strong correlation was found between [1-^13^C]lactate and [^13^C]bicarbonate, whereas [1-^13^C]pyruvate was only moderately correlated either image. The lactate-pyruvate (LPR) and bicarbonate-pyruvate (BPR) residual images were also strongly correlated with each other. The lactate-bicarbonate residual (LBR) was correlated with [1-^13^C]lactate and the LPR. The residual images are normalized to a whole brain mean of 0. All other images were normalized to a mean of 1. Points in scatter plots are individual voxels where the reverse grayscale colormap represents the density (i.e., darker colors indicate areas with more voxels).

**Fig. 2.**
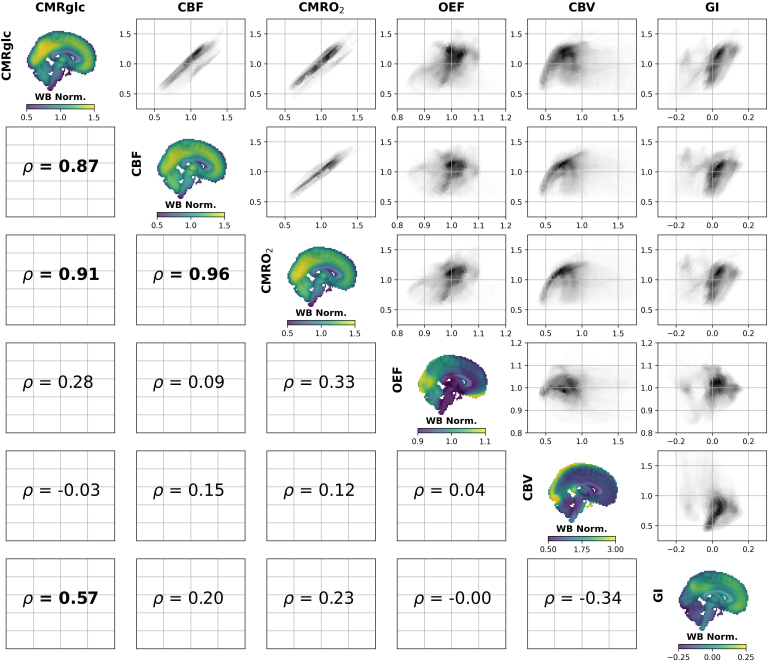
Comparison of spatial patterns of cerebral metabolism measured with PET. All conventions follow Figure 1. As was previously shown (Vaishnavi et al., 2010), the spatial patterns of cerebral glucose consumption (CMRglc), blood flow (CBF), and oxygen consumption (CMRO_2_) are very similar. The glycolytic index, an indicator of aerobic glycolysis (AG), was moderately correlated with CMRglc.

We first compared the group-averages images within modalities (Figs. 1 and 2). The strongest association within the HP ^13^C MRI dataset was between [1-^13^C]lactate and [^13^C]bicarbonate (ρ = 0.89, *p* *<* 0.0001; Fig. 1). To address the possibility that this correlation was driven by a common association with [1-^13^C]pyruvate (ρ ≥ 0.52, *p* < 0.01), we regressed each image against [1-^13^C]pyruvate to obtain pyruvate residual images (LPR and BPR). LPR and BPR were also highly correlated (ρ = 0.86, *p* < 0.001), and both residual images were strongly associated with their respective metabolite images before regression (ρ ≥ 0.88, *p* < 0.0001). The LBR was most strongly associated with [1-^13^C]lactate (ρ = 0.73, *p* < 0.0001) and the LPR (ρ = 0.66, *p* < 0.0001). As was previously shown (Vaishnavi et al., 2010), CMRglc, CBF, and CMRO_2_ are highly correlated (ρ ≥ 0.87, *p* < 0.0001; Fig. 2). There is also a moderate correspondence between GI and CMRglc (ρ = 0.57, *p* < 0.01).

**Fig. 3.**
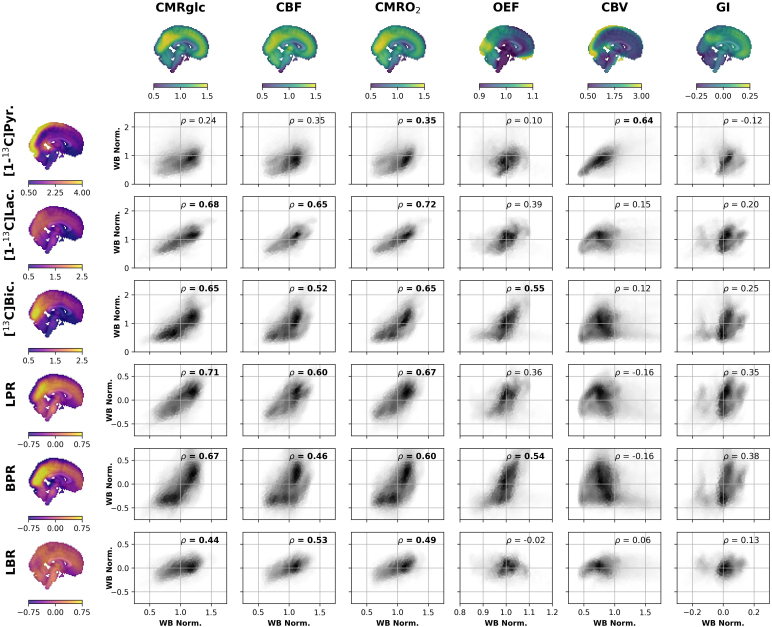
Between-modality comparison of regional cerebral metabolism and blood flow. Scatterplot matrix where rows are HP images, and columns PET images. Each cell of the matrix is a scatter plot with PET on the x-axis and HP on the y-axis. Correlations that are significantly different from zero are shown in bold (see section 2). [1-^13^C]lactate and [^13^C]bicarbonate were both correlated with glucose consumption (CMRglc), blood flow (CBF), and oxygen consumption (CMRO_2_). Neither parameter was strongly correlated was the glycolytic index (GI). Regressing the [1-^13^C]pyruvate topography from [1-^13^C]lactate (LPR) and [^13^C]bicarbonate (BPR) only slightly impacted their correlation with CMRglc, CBF, and CMRO_2_. The strongest correlations with OEF were [^13^C]bicarbonate and the BPR. [1-^13^C]pyruvate and CBV were strongly correlated, suggesting that vascular signal dominates the [1-^13^C]pyruvate image. All other conventions follow Figure 1.

Next, each HP ^13^C image was correlated with each PET image. Voxelwise comparisons are shown in Figure 3, and parcel-level correlations in Supplementary Figure S5. [1-^13^C]pyruvate was most strongly associated with CBV (ρ = 0.64; Fig. 3), a relationship that was statistically significant at the parcel level by spin permutation test (*p* < 0.001; Supplementary Fig. S5). [1-^13^C]pyruvate was also modestly correlated with CMRO_2_ (ρ = 0.35, *p* < 0.01). Both [1-^13^C]lactate and [^13^C]bicarbonate were significantly correlated with CMRglc, CMRO_2_, and CBF (ρ ≥ 0.52, p < 0.001), with the strongest correlation being between [1-^13^C]lactate and CMRO_2_ (ρ = 0.72, p < 0.0001). [^13^C]bicarbonate was also moderately correlated with OEF (ρ = 0.55, p < 0.01).

Regressing out the [1-^13^C]pyruvate signal from [1-^13^C]lactate and [^13^C]bicarbonate had a minimal effect on their correlations with CMRglc, CBF, CMRO_2_, and OEF (Fig. 3) and correlations that were significant without regression remained so with regression (Supplementary Fig. S5). Removing the [1-^13^C]pyruvate topography did moderately increase each metabolite’s correlation with GI, although the relationship was not significant for either the LPR (ρ = 0.35, *p* = 0.38) or BPR (ρ = 0.38, *p* = 0.76). The LBR correlated modestly with CMRglc, CBF, and CMRO_2_ (ρ ≥ 0.44, *p* < 0.01).

**Fig. 4.**
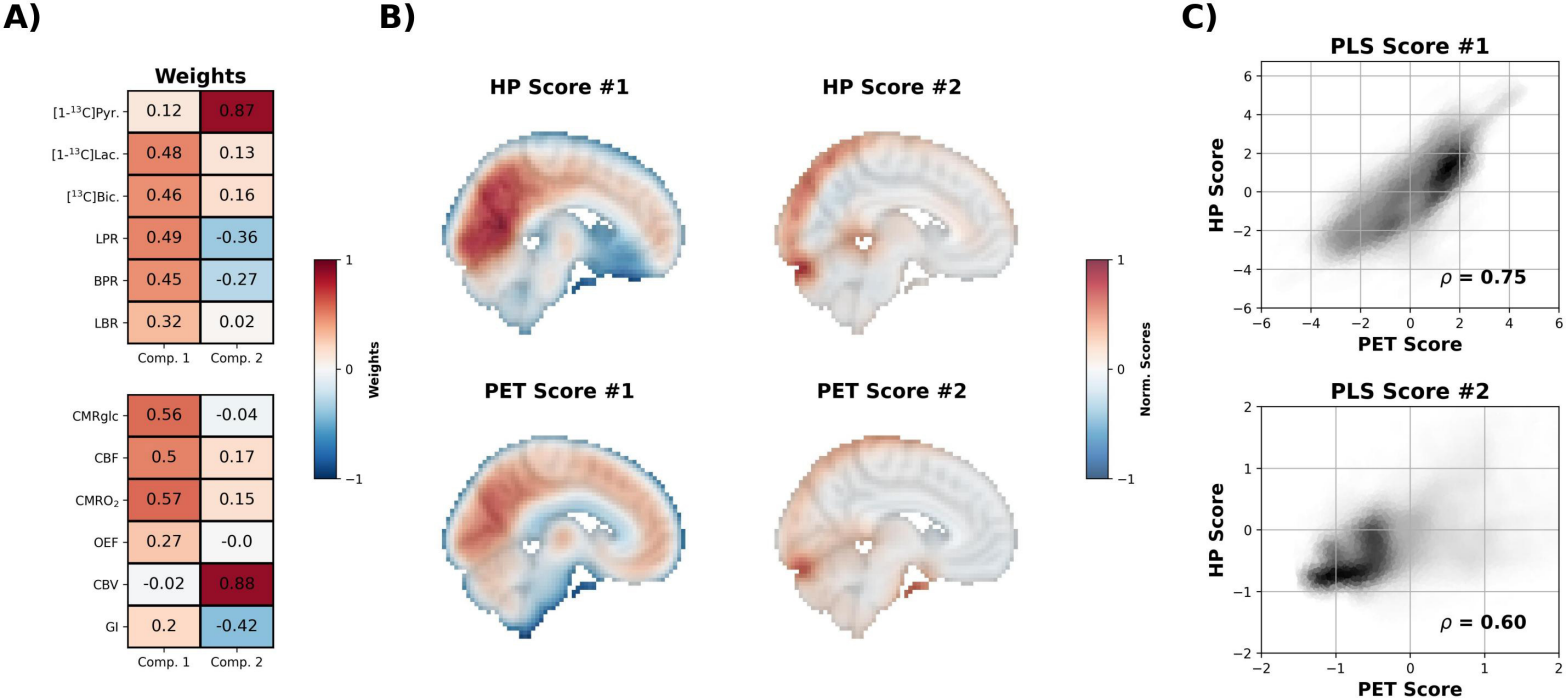
Partial least squares analysis of HP ^13^C MRI and metabolic PET images. Partial least squares was used to find the linear combination of HP and PET images which maximized the covariance between the two datasets. (A) The weight vectors for the first two components. The weights for the first component largely consisted of [1-^13^C]lactate [^13^C]bicarbonate, lactate-pyruvate residual (LPR), and bicarbonate-pyruvate residual (BPR) for the HP dataset, and glucose consumption (CMRglc), blood flow (CBF), and oxygen consumption (CMRO_2_) for PET. The second component was dominated by blood volume (CBV) for PET and [1-^13^C]pyruvate for HP (B) The scores for each component/dataset, normalized to a maximum of 1 for visualization purposes. (C) Scatter plots comparing each set of scores (each point is a voxel).

We then used PLS to identify the two components that maximized the variance between the HP ^13^C and PET images. The images that were given the largest weight in the first component were [1-^13^C]lactate, [^13^C]bicarbonate, LPR, and BPR for HP and CMRglc, CBF, CMRO_2_ for PET (Fig. 4A). The weights for the second component were dominated by CBV for PET and [1-^13^C]pyruvate for HP. The HP scores and PET scores were correlated with each other for both components (ρ ≥ 0.6; Fig. 4B & C).

## 4 Discussion

Although HP ^13^C MRI is a promising method for brain imaging, it is still in its infancy, with a recent review finding that only 25 published studies have used HP [1-^13^C]pyruvate to study human brain metabolism (Larson et al., 2024). As many of these studies focused on specific patient populations, it is not yet clear what mechanisms are responsible for HP [1-^13^C]pyruvate metabolism in the healthy human brain. To better understand the biological processes behind the HP ^13^C MRI signal, we compared the spatial distributions of injected [1-^13^C]pyruvate and its metabolites with the topographies of PET tracers of glucose consumption, oxygen metabolism, and blood flow. Using both pairwise correlations and PLS, we found that [1-^13^C]pyruvate was most strongly associated with CBV. This suggests a significant amount of [1-^13^C]pyruvate remains in the vascular compartment. On the other hand, [1-^13^C]lactate and [^13^C]bicarbonate correlated best with PET measurements of CMRglc, CBF, and CMRO_2_. This indicates that, at least in the healthy adult brain, [1-^13^C]lactate is more associated with overall metrics of energy metabolism than non-oxidative glucose consumption.

Our finding that the topography of [1-^13^C]pyruvate is highly correlated with CBV is consistent with previous findings of high [1-^13^C]pyruvate signal in the brain’s vasculature (Grist et al., 2019). It could also explain why the topography of [1-^13^C]pyruvate was only modestly correlated with [1-^13^C]lactate and [^13^C]bicarbonate, even though [1-^13^C]pyruvate inflow is required for the appearance of either metabolite. The presence of [1-^13^C]pyruvate in vascular structures is likely due to a combination of the low first-pass extraction fraction of pyruvate (~5 to 10% in rodents) (Miller & Oldendorf, 1986), and the short time window (~1 min) over which [1-^13^C]pyruvate images are acquired. Indeed, [^18^F]FDG, which has an extraction fraction of around 20% (Huisman et al., 2012), is largely confined to the vasculature in the first minutes following injection (Silvestri et al., 2022). As the [1-^13^C]pyruvate signal is much greater in the vasculature than in tissue, removing the vascular component from [1-^13^C]pyruvate images may improve tissue measurements. The vascular contribution in PET data is typically removed using either a kinetic model (Morris et al., 2004), or, as demonstrated here for [^15^O]O_2_, by regressing out the contribution of a subject-specific blood-volume image. Although not widely employed, it has been shown that vascular signal can be removed from HP ^13^C images of the kidney using flow-sensitive gradients (Gordon et al., 2016), or by administering a high molecular weight paramagnetic relaxation agent after the HP bolus (Reed et al., 2016). Applying these, or similar techniques to HP [1-^13^C]pyruvate brain images is an interesting direction for future research.

When [1-^13^C]pyruvate is converted to acetyl-CoA by pyruvate dehydrogenase (PDH), the labeled carbon is transferred to [^13^C]CO_2_, where it is converted to [^13^C]bicarbonate by carbonic anhydrase (CA). Since acetyl-CoA is the entry point of the citric acid cycle and the CA reaction occurs rapidly at normal pH levels (Tripp et al., 2001), [^13^C]bicarbonate production is generally considered to be a readout of oxidative metabolism. Our results are generally consistent with this view, with [^13^C]bicarbonate having a similar topography to both CMRO_2_ and OEF. Conversely, HP [1-^13^C]lactate production is often thought to reflect non-oxidative metabolism in the form of lactate production via lactate dehydrogenase (LDH) (Wang et al., 2019). Indeed, brain tumors, which overexpress LDHA (Valvona et al., 2016), have elevated [1-^13^C]lactate production (Autry et al., 2020; Miloushev et al., 2018; Park et al., 2010).

As the conversion of pyruvate to lactate is a component of AG, one might expect a regional correlation between AG and HP [1-^13^C]lactate production. However, in the healthy brain we found that the spatial distribution of [1-^13^C]lactate correlated more strongly with CMRO_2_ than GI. Furthermore, in our PLS analysis GI contributed more towards the second component, which was dominated by [1-^13^C]pyruvate, than the second component which had larger weights for [1-^13^C]lactate and [^13^C]bicarbonate. Although regressing out the contribution of regional differences in [1-^13^C]pyruvate signal from the [1-^13^C]lactate images did increase the correlation with GI (ρ of 0.20 to 0.35), it was still less than what was seen previously for CMRglc, CBF, and CMRO_2_ (ρ > = 0.65).

Several plausible explanations exist for why [1-^13^C]lactate does not correlate as strongly with GI as it does with other metrics. First, GI is a measure of total non-oxidative glucose consumption, not only lactate production. We have previously found that net lactate production does not account for all of AG in the brain (Blazey et al., 2018), leaving room for other mechanisms such as the pentose phosphate shunt and biosynthesis. Therefore, the topographical differences between GI and [1-^13^C]lactate could reflect regional differences in specific components of AG. The large dose of [1-^13^C]pyruvate used in human imaging experiments could also alter basal metabolism to such an extent that it reduces the correlation between GI and [1-^13^C]lactate. Assuming a total blood volume of 75 mL/kg (Oberholzer et al., 2024), a standard dose of [1-^13^C]pyruvate (0.43 mL/kg of a 250 mM solution) could raise the blood pyruvate concentration from the reference range of 0.060–0.145 mM (Kratz et al., 2004) to ~1.4 mM (Rodrigues et al., 2014). It is, therefore, reasonable to hypothesize that regional differences in [1-^13^C]lactate production might reflect regional differences in the ability to consume pyruvate (e.g., a high NADH/NAD^+^ ratio) more than regional differences in glycolysis.

Another potential explanation is that [1-^13^C]lactate production reflects, at least in part, biological processes other than AG. Recently, Rao et al. showed that transport through monocarboxylic transporter-1 limits the conversion of HP [1-^13^C]pyruvate to [1-^13^C]lactate in mice with xenografted tumors (Rao et al., 2020). Moreover, a study of 12 patients with prostate cancer revealed that [1-^13^C]lactate production is higher in tumors with elevated monocarboxylic transporter-1 (Granlund et al., 2020). Although these findings need to be replicated in the healthy brain, they suggest that pyruvate transport, and not pyruvate metabolism, is the primary factor controlling the [1-^13^C]lactate signal. The strong correlation we observed between blood flow and [1-^13^C]lactate is consistent with this view.

Alternatively, an influential early study by Day et al. showed that increasing the unlabeled lactate concentration in cultured cells increases the [1-^13^C]lactate signal, suggesting that the ^13^C label is rapidly exchanged between pyruvate and lactate, and therefore that the [1-^13^C]lactate signal reflects lactate concentration more than lactate flux (Day et al., 2007). The relationship between lactate concentration and [1-^13^C]lactate production was later replicated *in vivo* by Hurd et al., who found that [1-^13^C]lactate levels are elevated in rat kidney and liver when unlabeled lactate is injected along with HP [1-^13^C]pyruvate (Hurd et al., 2013). However, studies in both the rat (Bøgh et al., 2022) and human brain (Uthayakumar et al., 2024) found no significant differences in [1-^13^C]pyruvate levels after saturating the [1-^13^C]lactate resonance, although there was a trend level decrease in the human study. As RF saturation effectively eliminates the MR signal at the resonance of interest, one would expect that saturating [1-^13^C]lactate would reduce the [1-^13^C]pyruvate signal if a large portion of pyruvate is in exchange with lactate. Interestingly, saturating the [1-^13^C]lactate resonance does decrease the available [1-^13^C]pyruvate signal in a mouse model of lymphoma (Kettunen et al., 2010). Therefore, it is unclear to what extent endogenous lactate concentration determines [1-^13^C]lactate production in healthy human brain tissue. Comparisons between HP [1-^13^C]pyruvate imaging and ^1^H magnetic resonance spectroscopy would be helpful in clarifying this issue.

Finally, studies in humans (Uthayakumar et al., 2024; Zaidi et al., 2024) and animal models (Bøgh et al., 2022) have found that saturating the [1-^13^C]lactate resonance decreases the [^13^C]bicarbonate signal. One interpretation of these findings it that that some of the [1-^13^C]lactate produced from [1-^13^C]pyruvate enters oxidative pathways in the brain. The production of [^13^C]bicarbonate from [1-^13^C]lactate is consistent with the robust spatial correlation we found between [1-^13^C]lactate and [^13^C]bicarbonate, a result that was unchanged by adjusting for the spatial distribution of [1-^13^C]pyruvate. [1-^13^C]lactate oxidation could also explain why the topography of [1-^13^C]lactate correlated with CMRO_2_, and why regressing [^13^C]bicarbonate from [1-^13^C]lactate did not improve the correlation between [1-^13^C]lactate and GI. More broadly, [1-^13^C]lactate oxidation is consistent with the astrocyte-neuron lactate shuttle hypothesis, which proposes that lactate is produced from glucose in astrocytes and then shuttled to neurons where it is used as fuel for oxidative phosphorylation (Pellerin & Magistretti, 1994, 2012). In this case, [1-^13^C]pyruvate, not glucose, would be converted to [1-^13^C]lactate in astrocytes and then shuttled to neurons for oxidation. The astrocyte-neuron lactate shuttle is controversial (Dienel, 2019), however, and more work is needed to clarify its connection to HP [1-^13^C]pyruvate MRI. Moreover, although [1-^13^C]lactate and [^13^C]bicarbonate were highly correlated with each other, their correlations with PET were not identical. OEF was more strongly correlated with [^13^C]bicarbonate than [1-^13^C]lactate, whereas CBF had a stronger association with [1-^13^C]lactate. Additionally, regressing [^13^C]bicarbonate from [1-^13^C]lactate (LBR) reduced, but did not eliminate, the correlation between [1-^13^C]lactate and CMRO_2_. This would suggest that the relationship between [1-^13^C]lactate and oxidative metabolism is not entirely dependent on [^13^C]bicarbonate.

Our work has two primary limitations. First, all our comparisons were conducted at the population level, with a different set of participants making up each group. Although the two groups were matched for age and sex, we cannot exclude the possibility that other group-level differences may have impacted our results. For example, participants in PET study were instructed to keep their eyes closed, whereas no instruction was given to participants in the HP MRI study. Moreover, we unable to examine the correlation between HP [1-^13^C]pyruvate MRI and metabolic PET within individuals or within brain regions. Future within-participant comparisons could be facilitated by using MR-based measurements of CBF, CBV, and OEF. These MR techniques are validated against PET (Cho et al., 2021; Fan et al., 2016; Uh et al., 2011), do not expose participants to radiation, and can be performed during the same imaging session as a HP [1-^13^C]pyruvate scan. However, the physiological processes underlying MRI-based methods differ from those of PET-based methods, and regional discrepancies in CBF and CBV estimates between the two modalities have been noted (Heijtel et al., 2014; Uh et al., 2011; Zhang et al., 2014).

Second, all our analyses were correlative, and so any mechanistic inferences are speculative. Disentangling the relationship between HP [1-^13^C]pyruvate MRI and metabolic PET is especially difficult given the strong correlations within each modality. For example, CMRglc and CMRO_2_ are so tightly correlated with CBF that it is unclear if the correlation we observed between these two variables and [1-^13^C]lactate is due to transport, metabolism, or both. Studies employing manipulations are needed to elucidate which metabolic processes are responsible for HP [1-^13^C]pyruvate metabolism in the human brain. Studies in rodents have shown that high doses of isoflurane act as a vasodilator, increasing [1-^13^C]pyruvate levels, without changing [1-^13^C]lactate or [1-^13^C]bicarbonate (Hyppönen et al., 2021; Josan et al., 2013). However, in addition to increasing blood flow, isoflurane profoundly reduces glucose and oxygen cerebral metabolism (Alkire et al., 1997; Oshima et al., 2003), making it difficult to separate transport from demand. An alternative is hypercapnic challenges, which increase blood flow without changing CMRglc (Villien et al., 2014).

Sensory stimulation, which focally increases CMRglc more than CMRO_2_ (Fox et al., 1988), could be used to assess how much non-oxidative metabolism drives production of [1-^13^C]lactate and [^13^C]bicarbonate. Although there have been two studies that have used HP [1-^13^C]pyruvate during visual stimulation, they have yielded conflicting results. One study found an increase in [1-^13^C]lactate without a corresponding increase in [^13^C]bicarbonate (Uthayakumar et al., 2025),^,^ while the other reported an increase in [^13^C]bicarbonate with no change in [1-^13^C]lactate (Zaidi et al., 2024). Both studies, however, are at odds with the strong correlation we found between [^13^C]bicarbonate and [1-^13^C]lactate, suggesting the increased metabolic demand imposed by sensory stimulation may alter [1-^13^C]pyruvate metabolism.

## 5 Conclusion

This study is an important initial step toward understanding how HP [1-^13^C]pyruvate MRI relates to well-established PET measurements of brain metabolism. We have shown that in the heathy human brain [1-^13^C]pyruvate is heavily influenced by CBV and that the topographies of [1-^13^C]lactate and [1-^13^C]bicarbonate are similar to those of CMRglc, CBF, and CMRO_2._

## Supplementary Material

Supplementary Material

## Data Availability

The group average HP and PET images, as well as the scripts necessary to reproduce each figure, can be found at: https://doi.org/10.5281/zenodo.14927726.
