# Supplementary material for: Spatial distribution of hyperpolarized [1-13C]pyruvate MRI and metabolic PET in the human brain
Source: Imaging Neurosci (Camb). 2025 Sep 26;3:IMAG.a.903. doi: 10.1162/IMAG.a.903 (PMC12477642; doi:10.1162/IMAG.a.903)
Supplement: Supplementary Material [file IMAG.a.903_supp.pdf]

Supplementary Information

Spatial distribution of hyperpolarized [1-13C]pyruvate MRI and metabolic PET in the human brain

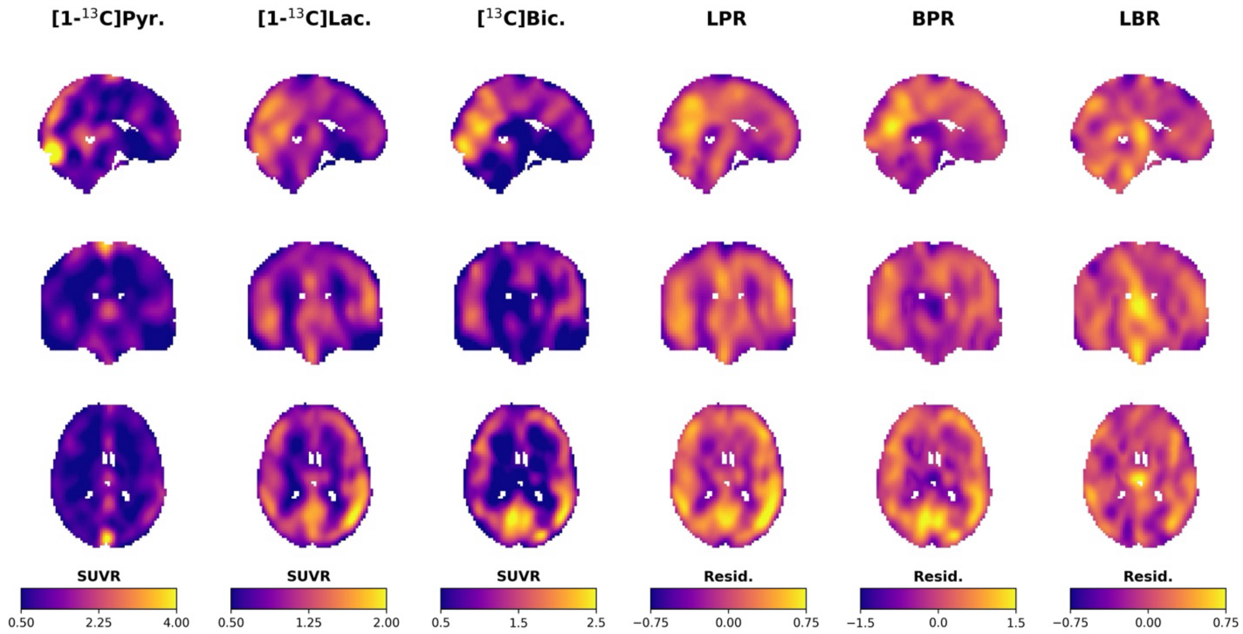

Supplementary Figure 1: Example HP ^{13}C MRI images from a randomly chosen participant. [1-^{13}C]pyruvate, [1-^{13}C]lactate, and [^{13}C]bicarbonate images were obtained by summing across time and then normalizing the resulting image to have a whole-brain mean of 1. The lactate-pyruvate (LPR), bicarbonate-pyruvate (BPR), and lactate-bicarbonate (LBR) residual images were created using spatial regression (**Supplementary Figure 3**).

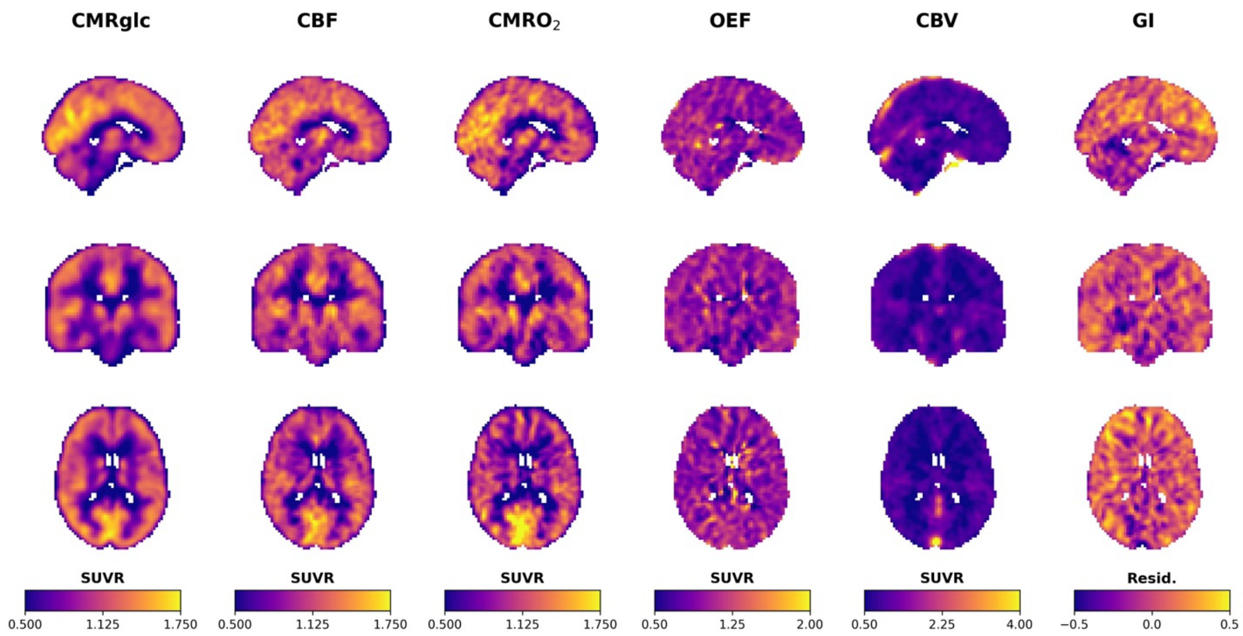

Supplementary Figure 2: Example metabolic PET images from a randomly chosen participant. CMRglc, CBF, $CMRO_2$, and CBV images were obtained by temporal summation of the dynamic PET data followed by normalization to the whole brain mean. OEF, or oxygen extraction fraction, is the ratio of blood flow and oxygen consumption (see Methods). The glycolytic index (GI) was created using spatial regression between CMRglc and $CMRO_2$.

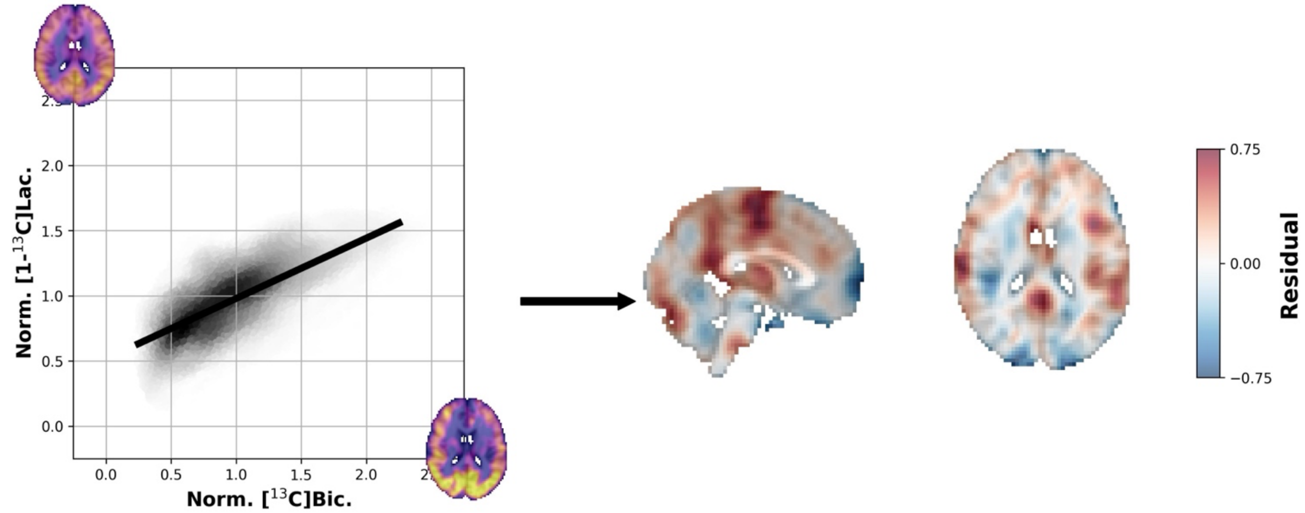

Supplementary Figure 3: A linear regression between [1-^{13}C]lactate and [^{13}C]bicarbonate images is used to compute a lactate-bicarbonate residual image for each participant (LBR; right). Prior to regression, each metabolic map was smoothed with a 15 FWHM gaussian kernel. Residuals were computed by applying the estimated linear coefficients to the unsmoothed data. Positive residual values indicate regions where relative lactate metabolism exceeds relative bicarbonate metabolism, whereas lower values indicate regions with higher relative bicarbonate production. The same procedure was used to produce the lactate-pyruvate (LPR) and bicarbonate-pyruvate (LBR) residual images.

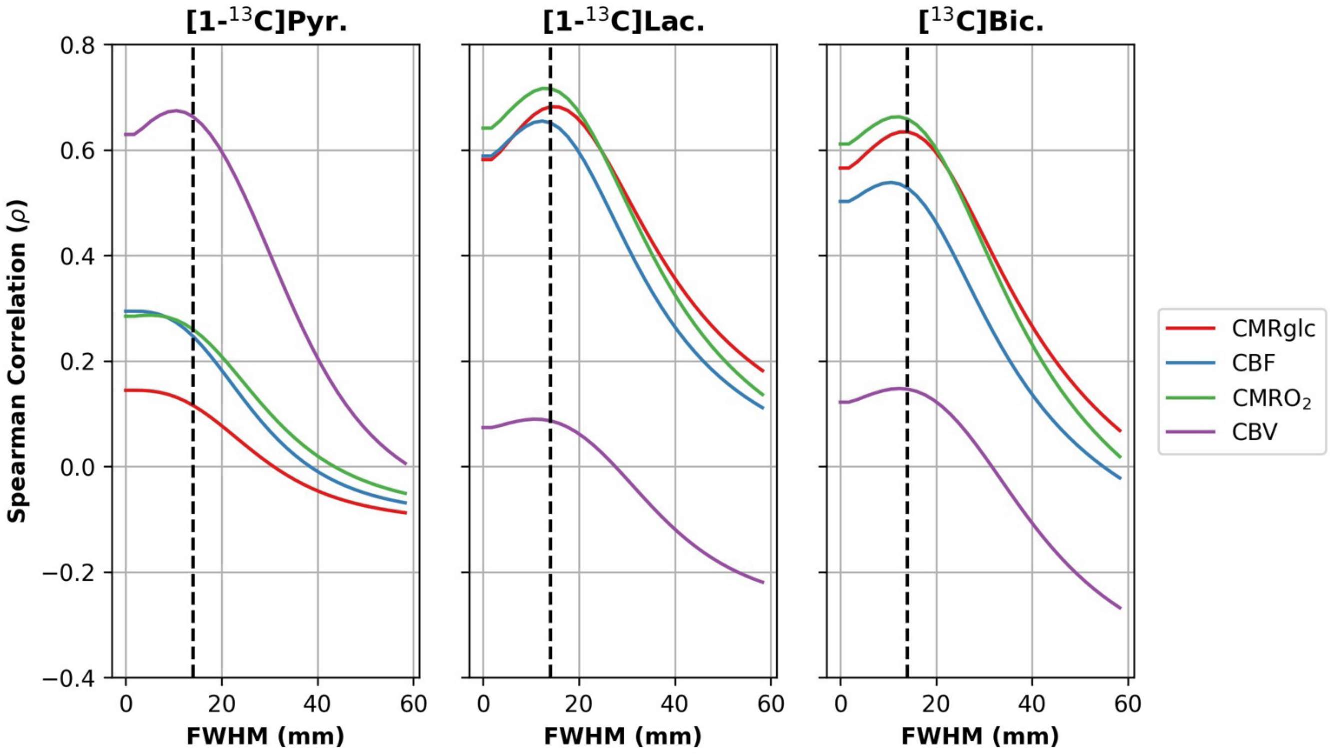

Supplementary Figure 4: The size of the smoothing kernel applied to the PET images was determined by measuring the correlation between the HP and PET images over a range of kernel sizes. A kernel around 14 mm FWHM (dashed black line) maximized the correlation across a range of comparisons. Note that while the kernel size had a large impact on the magnitude of the correlation, it had less of an effect on the rank order of the correlations within each HP image.

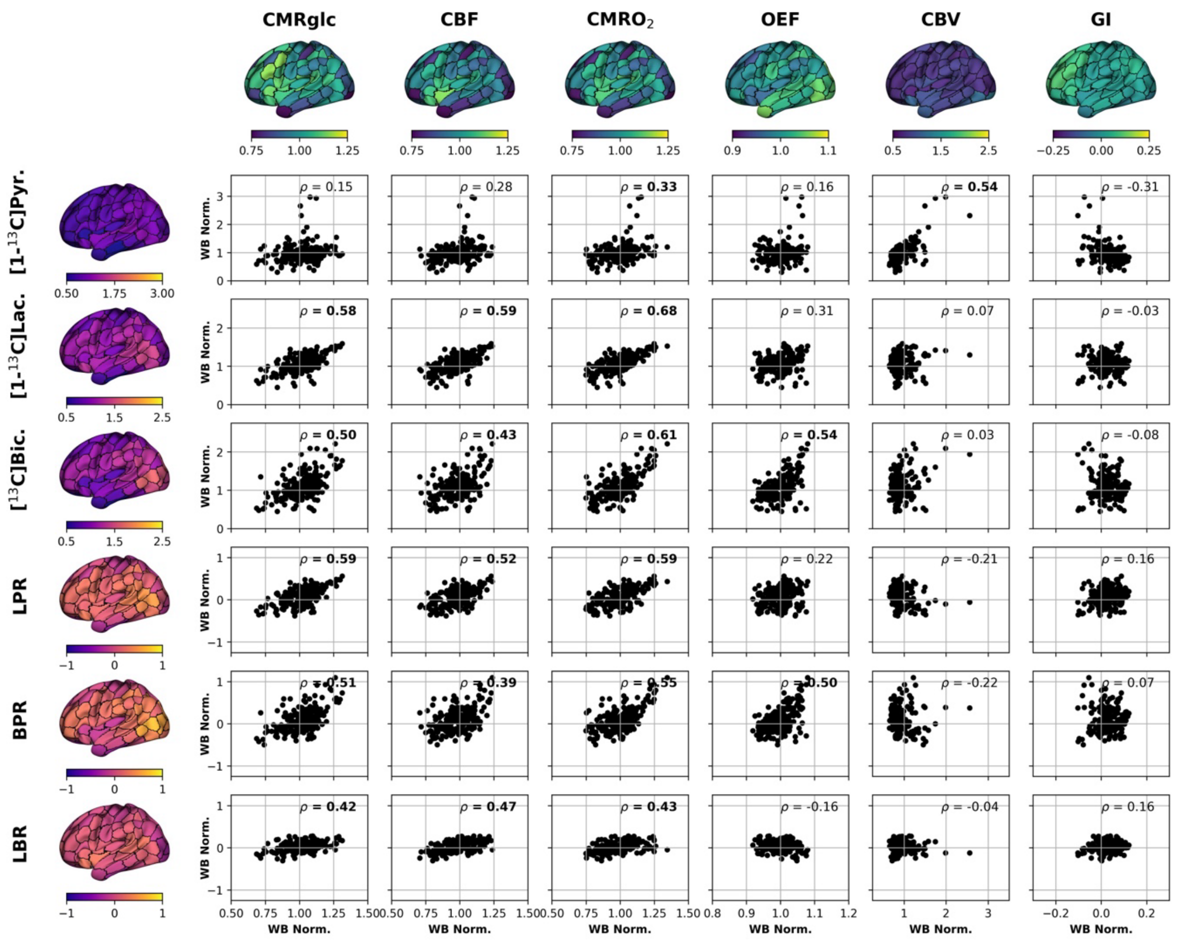

Supplementary Figure 5: Between-modality comparison of regional cerebral metabolism within the cerebral cortex. Scatterplot matrix where rows are HP images, and columns PET images. Each cell of the matrix is a scatter plot with PET on the x-axis and HP on the y-axis. Each point in the scatterplot is a cortical parcel defined in the Schaefer 7-network 200 region atlas[1]. Spearman correlations in bold are significantly different ($p < 0.05$; false discovery rate corrected) from zero based on a spin-permutation test[2,3]. [1-^{13}C]lactate and [^{13}C]bicarbonate were correlated with glucose consumption (CMRglc), oxygen metabolism ($CMRO_2$), and blood flow (CBF), before and after regressing out [1-^{13}C]pyruvate (LPR and BPR). [1-^{13}C]lactate also remained correlated with CMRglc, $CMRO_2$, and CBF after regression with [^{13}C]bicarbonate (LBR). [^{13}C]bicarbonate and the BPR were also correlated with the oxygen extraction fraction (OEF). Only [1-^{13}C]pyruvate was correlated with blood volume (CBV). No images were significantly correlated with the glycolytic index (GI).

References

1. Schaefer A, Kong R, Gordon EM, et al. Local-Global Parcellation of the Human Cerebral Cortex from Intrinsic Functional Connectivity MRI. *Cereb Cortex* 2018; 28: 3095–3114.
2. Alexander-Bloch AF, Shou H, Liu S, et al. On testing for spatial correspondence between maps of human brain structure and function. *Neuroimage* 2018; 178: 540–551.
3. Benjamini Y, Yekutieli D. The control of the false discovery rate in multiple testing under dependency. *The Annals of Statistics* 2001; 29: 1165–1188.
